# GM-1020: a novel, orally bioavailable NMDA receptor antagonist with rapid and robust antidepressant-like effects at well-tolerated doses in rodents

**DOI:** 10.1038/s41386-023-01783-1

**Published:** 2024-01-04

**Authors:** Adam K. Klein, Eric W. Austin, Michael J. Cunningham, Dino Dvorak, Silvia Gatti, Sarah K. Hulls, Laszlo Kiss, Andrew C. Kruegel, Gerard J. Marek, Mariusz Papp, Jonathan Sporn, Zoë A. Hughes

**Affiliations:** 1 Gilgamesh Pharmaceuticals, New York, NY USA; 2 McArthur and Associates, Basel, Switzerland; 3 Maj Institute of Pharmacology, Polish Academy of Sciences, Krakow, Poland

**Keywords:** Pharmacology, Depression, Ion channels in the nervous system

## Abstract

The NMDA receptor (NMDAR) antagonist ketamine has shown great potential as a rapid-acting antidepressant; however, its use is limited by poor oral bioavailability and a side effect profile that necessitates in-clinic dosing. GM-1020 is a novel NMDAR antagonist that was developed to address these limitations of ketamine as a treatment for depression. Here, we present the preclinical characterization of GM-1020 alongside ketamine, for comparison. In vitro, we profiled GM-1020 for binding to NMDAR and functional inhibition using patch-clamp electrophysiology. In vivo, GM-1020 was assessed for antidepressant-like efficacy using the Forced Swim Test (FST) and Chronic Mild Stress (CMS), while motor side effects were assessed in spontaneous locomotor activity and on the rotarod. The pharmacokinetic properties of GM-1020 were profiled across multiple preclinical species. Electroencephalography (EEG) was performed to determine indirect target engagement and provide a potentially translational biomarker. These results demonstrate that GM-1020 is an orally bioavailable NMDAR antagonist with antidepressant-like efficacy at exposures that do not produce unwanted motor effects.

## Introduction

The discovery of the rapid antidepressant effects of the NMDA receptor (NMDAR) antagonist (*rac*)-ketamine ushered in a new wave of interest in identifying molecules that can achieve the rapid and robust efficacy of (*rac*)-ketamine but improve on some of its shortcomings [1, 2]. At the commonly administered antidepressant dose of (*rac*)-ketamine (0.5 mg/kg, i.v. infusion over 40 min), patients experience side effects of dissociation, cognitive impairment, sedation, and ataxia, which restrict safe administration to a supervised, in-clinic setting, thereby limiting the clinical uptake of this treatment [3]. Similarly, preclinical characterization of ketamine indicates that it causes antidepressant-like effects in rodents at doses overlapping those that cause motor impairment and cognitive disruption [4–8]. In addition, (*rac*)-ketamine has poor oral bioavailability that necessitates parenteral dosing. Spravato^®^ (esketamine), an intranasal formulation of the more potent *S*-enantiomer of ketamine, has been approved for patients with treatment-resistant depression, but still requires supervised administration under a restrictive Risk Evaluation and Mitigation Strategy (REMS) program due to sedative and dissociative effects [9].

A pilot open-label study with intravenous *R*-ketamine indicates that the less potent enantiomer retains antidepressant efficacy at doses that do not cause dissociation, suggesting an improved therapeutic index (TI) is possible for this mechanistic class [10]. Preclinical data support this improvement in TI [11–14]. However, there remains a need for a non-dissociative, orally bioavailable NMDAR antagonist with rapid and robust antidepressant efficacy. Here, we present the preclinical characterization of GM-1020 ((R)-2-(4-fluorophenyl)-2-(methylamino)cyclohexan-1-one): an analog of ketamine that is an orally bioavailable, NMDAR antagonist with robust, durable antidepressant-like effects and a greater separation from motor effects in rodents.

## Materials and methods

Additional technical details, materials, experimental procedures, and statistical methods are in the Supplementary Methods.

### In vitro pharmacology

The affinity for NMDAR was determined using [^3^H]MK-801 radioligand binding in rat cerebrum homogenate as previously described [15, 16]. A comparison of the inhibition of glutamate/glycine currents in oocytes expressing human NR1/NR2A or NR1/NR2B NMDAR was made using patch clamp as previously described [17]. Patch clamp electrophysiology studies to investigate concentration and voltage dependence of NMDAR currents were conducted in HEK293 NMDA NR1/2A TET-inducible cells as previously described [18, 19].

Binding affinity of GM-1020 at µ-opioid receptor (MOR) using [^3^H]DAMGO and MOR agonist activity measurements (FLIPR Ca^2+^ mobilization) were conducted as previously described [20, 21]. The off-target pharmacology of GM-1020 (10 µM) was assessed using a broad panel of in vitro receptor, enzyme, or transporter assays (Cerep SafetyScreen87 panel).

### In vivo pharmacology

#### Animals

Unless otherwise specified, all experiments used adult male C57BL6/J mice housed 2–5/cage, or adult male Sprague-Dawley rats housed 2/cage (see Supplementary Methods for precise ages). All animals had *ad libitum* access to food and water and were maintained in a light and temperature-controlled vivarium. All studies with live animals were approved by the ethics committee at the institution where they took place and conform to the standards set forth in the NIH Guide for Care and Use of Laboratory Animals.

#### Forced Swim Test (FST)

A modified version of the previously described FST was used [22, 23]. After a 15 min pre-swim rats were administered vehicle or GM-1020 (1-32 mg/kg; s.c.). Immobility time was measured during a 5 min swim test 23.5 h post dose. An additional group of rats was dosed with the positive control, desipramine (20 mg/kg, s.c.) three times (23.5 h, 4 h and 0.5 h) prior to the swim test.

#### Chronic Mild Stress (CMS)

The first CMS study was carried out in male Wistar–Han rats as previously described [24–28], with the experimental schedule shown in Fig. 2B. For each treatment group *n* = 8 rats were exposed to CMS stressors, and *n* = 8 rats were unstressed controls. A second CMS study was conducted to evaluate efficacy of GM-1020 after oral dosing and the duration of efficacy following a single dose in male Wistar-Kyoto rats (Charles River, Sulzfeld, Germany). Animals in both studies were single housed prior to the onset of stress. The EPM and NOR tests were not included in the second study.

#### Studies of motor effects

The effects of GM-1020 (3.2–32 mg/kg; s.c.) on motor function were compared to those of (*rac*)-ketamine (3.2–32 mg/kg; s.c.) in rats. Spontaneous locomotor activity (sLMA) was monitored for 30 min pre-dose and 60 min post-dose. Motor coordination was assessed 5 min after dosing using an accelerating rotarod. Plasma samples were collected from satellite rats to determine concentrations of GM-1020 or *(rac)-*ketamine 30 or 18 min after dosing, respectively. GM-1020 (1–32 mg/kg; s.c.) was also assessed in mice in both sLMA and the rotarod.

#### Conditioned place preference (CPP)

The effects of GM-1020 (1–32 mg/kg; s.c.) on CPP were compared to oxycodone (3 mg/kg; s.c.) in mice.

#### EEG Studies

The effects of GM-1020 (1-10 mg/kg, s.c.) on EEG were measured using a within-subjects design in a cohort of 8 freely moving rats implanted with 2 cortical electrodes connected to a DSI transmitter as previously described [29].

#### Pharmacokinetic (PK) studies

Oral bioavailability of GM-1020 was determined by comparing PK 0-24 h after i.v. and p.o. administration (1 mg/kg) in multiple preclinical species. Oral bioavailability of *(rac)-*ketamine (10 mg/kg) was also determined in rat. Brain and plasma PK were measured in rats after administration of GM-1020 (10 mg/kg, p.o.). In vitro ADME was conducted as previously described [30].

### Statistical analysis

All statistical tests were conducted using GraphPad Prism (9 or 10). Post-hoc testing using Šídák’s multiple comparisons corrections was used when comparing all pairs of means and Dunnett’s test was used when comparing each treatment condition to a vehicle-treated control group. Further statistical details can be found in the supplementary methods. The results of the statistical tests are described in the corresponding figure legend.

## Results

### NMDAR binding affinity

**Fig. 1:**
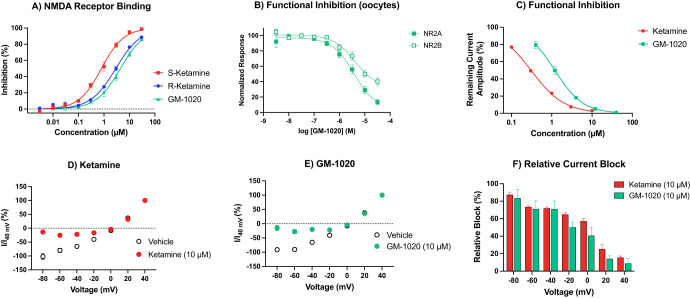
In vitro pharmacology of GM-1020 at NMDA receptors. **A** Displacement of [^3^H]MK-801 binding by GM-1020, *S*-ketamine, and *R*-ketamine in rat brain homogenate (*n* = 2-3/concentration). *K*_i_ values were determined to be 3.25, 0.56, and 2.05 µM, respectively. **B** Potency of GM-1020 to inhibit glutamate/glycine induced currents in oocytes expressing NR2A or NR2B containing NMDA receptors. The IC_50_ of GM-1020 at NR2A was 3.70 and NR2B 4.16 µM (*n* ≥ 5 cells/concentration). **C**–**F** Results of patch clamp experiments in HEK293 cells expressing human NR2A containing NMDAR (*n* ≥ 5 cells/concentration). **C** Concentration response for (*rac*)-ketamine (IC_50_: 0.305 µM) and GM-1020 (IC_50_: 1.19 µM) on inhibition of the current amplitude. **D**, **E** Voltage dependent responses of (*rac*)-ketamine and GM-1020, respectively. For (*rac*)-ketamine, a two-way ANOVA revealed significant main effects of both group (F(1, 56) = 167.0, *p* < 0.0001) and voltage (F(6, 56) = 347.6, *p* < 0.0001), and the interaction between the terms (F(6, 56) = 30.99, *p* < 0.0001). Post-hoc testing with Šídák’s multiple comparisons test revealed significant differences between vehicle and ketamine at −80, −60, −40 (*p* < 0.0001), and −20 mV (*p* = 0.0024). For GM-1020, a two-way ANOVA revealed significant main effects of both group (F(1, 42) = 164.2, *p* < 0.0001) and voltage (F(6, 42) = 370.2, *p* < 0.0001), and the interaction between the terms (F(6, 42) = 28.66, *p* < 0.0001). Post-hoc testing with Šídák’s multiple comparisons test revealed significant differences between vehicle and GM-1020 at −80, −60, −40 (*p* < 0.0001), and -20 mV (*p* = 0.0209). **F** Relative current block by (*rac*)-ketamine (10 µM, red bars) and GM-1020 (10 µM, green bars). A two-way ANOVA revealed a significant main effect of group (F(1, 49) = 8.021, *p* = 0.0067), a significant main effect of voltage (F(6, 49) = 53.62, *p* < 0.0001), and no significant interaction (F(6, 49) = 0.6715, *p* = 0.6731). Post-hoc testing revealed there was no significant difference between (*rac*)-ketamine and GM-1020 at any voltage. All figures depict the mean ± SEM.

GM-1020 displaced [^3^H]MK-801 binding to NMDAR in rat cortical tissue with low-micromolar affinity (*K*_i_ 3.25 µM; 95% CI 2.6–3.7 µM), while *R*-ketamine and *S*-ketamine had binding affinities of 2.05 (95% CI 1.8–2.3 µM) and 0.56 µM (95% CI 0.49–0.64 µM), respectively (Fig. 1A).

### Functional inhibition in oocytes

GM-1020 showed functional inhibition of NMDAR-mediated currents in NR2A- and NR2B-containing receptors, with IC_50_ values of 3.70 µM and 4.16 µM, respectively (Fig. 1B). This demonstrates that GM-1020 has similar antagonist potency at NR2A- and NR2B-containing NMDAR.

### Functional inhibition in HEK cells

Both *(rac)*-ketamine and GM-1020 showed functional inhibition of NR1/2A-NMDAR-mediated currents, with IC_50_ values of 0.305 µM and 1.192 µM, respectively (Fig. 1C). Both compounds showed voltage-dependent block of NR1/2 A receptors, with greater current block at more negative voltages, and no effect at positive voltages (Fig. 1D, E). The observed voltage dependence did not differ between GM-1020 and ketamine (Fig. 1F). Like ketamine, GM-1020 is an NMDAR antagonist that inhibits the receptor in a voltage-dependent manner.

### Off-target pharmacology

Arylcyclohexylamines like ketamine have weak affinity for MOR, (K_i_ *S*-Ketamine: 7 µM; *R*-ketamine: 19 µM) [11]. GM-1020 was evaluated in HEK cells stably transfected with human MOR and *G*_α15_ to facilitate promiscuous coupling of MOR to calcium release. GM-1020 had a binding affinity (*K*_i_) of 19 µM in [^3^H]DAMGO competition binding and no agonist activity up to 100 µM (DAMGO *K*_i_ 0.09 nM, EC_50_ 3.94 nM, *E*_max_ 95%).

To further understand its NMDAR selectivity, GM-1020 (10 µM) was profiled in the Cerep87 SafetyScreen panel (Table S5). As expected, GM-1020 competed for the phencyclidine binding site of NMDAR. Follow-up competition binding studies for all off-target hits with >15% inhibition revealed GM-1020 affinities (*K*_i_) of >1 mM for AMPA, melanocortin 4, and α_4_β_2_ nicotinic acetylcholine receptors, and 6.2 µM for the serotonin transporter. A follow-up cAMP assay revealed no agonist activity at the 5-HT_1B_ receptor (EC_50_ > 10 µM, *E*_max_ −3.73%).

### In vitro ADME

GM-1020 showed high stability in liver microsomes (human Cl_int(mic)_ 7.7 µL/min/mg). Microsomal clearance values for preclinical species are shown in Table 1. Microsomal clearance values were used to predict hepatic clearance (Cl_int(liv)_, mL/min/kg) in each species. Since GM-1020 was found to be largely unbound to plasma (21-25%), and assuming GM-1020 first pass hepatic clearance as the limiting determinant of oral bioavailability, oral bioavailability (%F) was predicted for each species using Cl_int(liv)_ and the published hepatic liver blood flow (Q_h_) [31] according to the equation: %F = 100*(1–Cl_int(hep)_/Q_h_). Microsomal clearance in vitro was found to be a good predictor of oral bioavailability in non-clinical species (%F; 28-85%) and predicted human oral bioavailability to be ~60%.

**Table 1:** Pharmacokinetic properties of GM-1020.

Plasma PK of GM-1020 1 mg/kg											
Compound	Species	Dose route	*T*_max_ (h)	*C*_max_ (ng/ml)	AUC _(0-t)_ (ng*h/ml)	*T*_*1*/2_ (h)	Cl_int_ (µL/min/mg)	Microsomal Clearance (% liver blood flow)	%*F* (predicted)		%*F* (actual)
GM-1020	Mouse, C57BL/6	Oral	0.25	53.6	38.8	0.49	13.2	15	85		40.3
		IV		299.3	96.3	0.75					
	Rat, SD	Oral	0.50	17.7	50.3	1.58	37.2	67	33		30.9
		IV		141.4	163.0	2.77					
(rac)-ketamine (10 mg/kg)	Rat, SD	Oral	0.17	189.5	121.6	0.56	128.0	232	0		9.1
		IV		3,892.5	1,335.0	0.69					
GM-1020	Dog, beagle	Oral	0.25	2.4	1.7	1.10	73.5	238	0		0.8
		IV		227.7	214.7	0.68					
	Minipig, bama/Göttingen	Oral	0.83	92.9	234.7	4.03	15.7	36	64		42.7
		IV		409.7	555.9	2.54					
	Cynomolgus	Oral	0.75	115.6	255.4	1.58	31.4	72	28		26.9
		IV		351.3	955.9	3.70					
	Human	–	–	–	–	–	7.7	37	63		–

Brain & Plasma PK of GM-1020 10 mg/kg, p.o.											
Compound	Species	Matrix	*T*_max_ (h)	*C*_max_ (ng/mL)	AUC_(0-t)_ (ng h/mL)	*T*_1/2_ (h)	Unbound *C*_max_ (ng/mL)	Unbound AUC_(0-t)_ (ng h/mL)		Unbound (*C*_max_) brain: plasma	
GM-1020	Rat, SD	Plasma	0.5	850.3	1200.7	1.17	552.7	780.5		1.32	
		Brain	0.5	2599.1	3787.8	1.18	727.8	1060.6			

Table provides a summary of the in vivo pharmacokinetics (PK) of GM-1020 dosed p.o. and i.v. in mouse, rat, dog, minipig, and cynomolgus monkeys. The PK of (rac)-ketamine (10 mg/kg p.o. and i.v.) in rats is also shown. The *in* vitro microsomal clearance as a percentage of blood flow for these species, as well as human, are also shown. %*F* values were predicted based on microsomal clearance (Cl_int(liv)_) and liver blood flow (Q_h_) for each species [31] according to the equation: %*F* = 100*(1 – Cl_int(hep)_/Q_h_). Brain PK of GM-1020 in rats after administration of 10 mg/kg (p.o.) is also included.

### Pharmacokinetics

The pharmacokinetic properties of GM-1020 are summarized in Table 1. GM-1020 demonstrated good oral bioavailability in non-clinical species other than dogs, with %*F* ranging from 27–43%. Importantly, except for mouse, predicted %F values closely aligned with measured %*F* across species, and human was predicted ~63%, providing confidence GM-1020 will exhibit high oral bioavailability in humans. The %F for ketamine in rats was only 9%. GM-1020 had a half-life ranging from ~40 min in dogs to 3.7 h in cynomolgus monkeys after i.v. dosing of 1 mg/kg. GM-1020 showed excellent brain penetration in rats dosed orally, with a free brain concentration to free plasma concentration ratio of 1.3.

To understand the exposures of GM-1020 achieved in the behavioral studies, we also measured PK in rats after dosing with 3 mg/kg s.c., 1.5 mg/kg i.p, and 10 mg/kg p.o. (Table S6). GM-1020 reached approximately the same *C*_max_ after both 3 mg/kg s.c. and 10 mg/kg p.o. (341.6 and 256.5 ng/mL, respectively), as well as a similar total exposure (AUC: 603.8 and 754.0 ng h/mL, respectively). The intraperitoneal dose of 1.5 mg/kg reached approximately half the *C*_max_ (151.1 ng/ml) with a much lower AUC (167.4 ng h/ml) compared to the subcutaneous (3 mg/kg) and oral (10 mg/kg) routes.

### Forced Swim Test (FST)

**Fig. 2:**
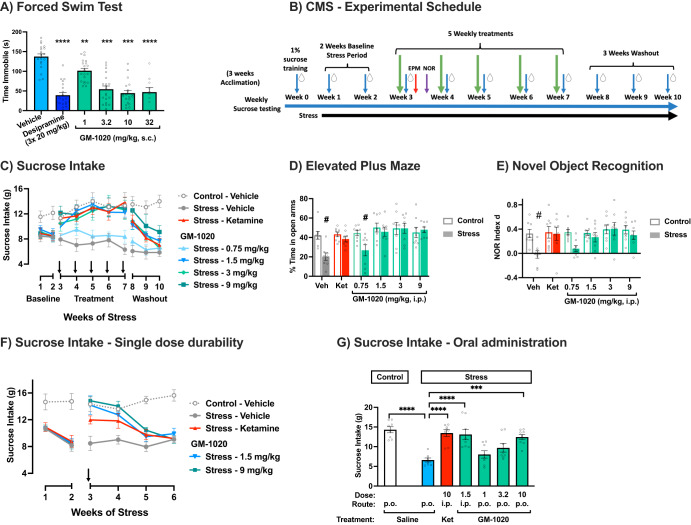
Antidepressant-like efficacy of GM-1020. **A** Effect of acute administration of GM-1020 (1-32 mg/kg, s.c.) on immobility time in naive rats tested in the FST 24 h after dosing (*n* ≥ 10/group). A one-way ANOVA revealed a significant main effect of treatment (F(5, 104) = 30.19, *p* < 0.0001). Asterisks indicate a significant difference from the vehicle treated group using Dunnett’s post-hoc test. The active comparator, desipramine (20 mg/kg, s.c.), dosed 3 times (23.5, 4 and 0.5 h) prior to testing also decreased immobility time. **B**–**E** Data collected in the first CMS study in Wistar Han rats (*n* = 8/group). **B** An overview of the experimental schedule for CMS studies. Blue arrows and drops represent weekly sucrose drinking tests (Tuesdays). Green arrows show when animals received weekly treatments (Mondays). The red arrow shows the timing for the EPM (Wednesday), and the purple arrow for the NOR test (Thursday). **C** Effect of GM-1020 (0.75–9 mg/kg, i.p.) and (*rac*)-ketamine (10 mg/kg, i.p.) on sucrose intake. Weeks 1 and 2 show the effect of CMS on sucrose intake prior to the initiation of drug treatment. During weeks 3–7, animals were administered GM-1020, (*rac*)-ketamine (as a positive control), or vehicle 24 h prior to measuring sucrose intake. During weeks 8–10, drug treatment was terminated while the CMS paradigm continued to test the durability of the antidepressant-like effect. A Mixed Model GLM with repeated measures was conducted on the data collected during weeks 3–10 (treatment duration and washout) from stressed animals, which revealed a significant main effect of Trial (F(7, 306.9) = 10.508, *p* < 0.0001), and a significant effect of Treatment (F(5, 70.2) = 8.31, *p* < 0.0001). Post-hoc testing with Šídák’s multiple comparisons revealed that only the group treated with 0.75 mg/kg of GM-1020 failed to differentiate from vehicle (F(1, 71.1) = 2.2499, *p* = 0.13). **D** Percent time spent in the open arms in the Elevated Plus Maze (EPM), which was conducted after the first dose, 24 h after sucrose intake measurement (48 h after treatment). Open bars show data from unstressed control rats, while filled bars are rats exposed to CMS. A two-way ANOVA revealed a significant main effect of Treatment (F(5, 84) = 5.591, *p* = 0.0002), a significant main effect of Stress (F(1, 84) = 9.126, *p* = 0.0033) and a significant interaction (F(5, 84) = 2.568, *p* = 0.0327). Post-hoc testing with Šídák’s multiple comparisons test revealed significant differences between stressed and unstressed groups treated with vehicle (*p* = 0.0040) and 0.75 mg/kg of GM-1020 (*p* = 0.0315). **E** Results from the Novel Object Recognition task (NOR). The NOR was conducted 72 h after the first dose. Open bars show data from unstressed control rats, while filled bars are rats exposed to CMS. A two-way ANOVA revealed a significant main effect of Treatment (F(5, 84) = 2.988, *p* = 0.0157), a significant main effect of Stress (F(1, 84) = 9.464, *p* = 0.0028) but no significant interaction (F(5, 84) = 1.834, *p* = 0.1150). Post-hoc testing with Šídák’s multiple comparisons test revealed significant differences between stressed and unstressed groups treated with vehicle (*p* = 0.0090). **F**, **G** Effects of (*rac*)-ketamine or GM-1020 on sucrose intake in WKY rats (*n* = 8/group). **F** Effect of a single administration of drug in week 3; sucrose intake was assessed 24 h later and then once weekly for a total of 4 weeks post dose. A two-way ANOVA revealed a significant main effect of group (F(4,35) = 12.34, *p* < 0.0001), time (F(2.456, 85.94) = 12.34, *p* < 0.0001), and a significant interaction (F(12, 105) = 4.334, *p* < 0.0001). Post hoc testing with Dunnett’s test showed that only stressed vehicle animals significantly different from unstressed vehicle animals on week 3 (*p* = 0.006) and week 4 (*p* = 0.0004). By week 5, all stressed animals were significantly different from unstressed vehicle animals (*p’s* < 0.001). **G** Effect of GM-1020 (1–10 mg/kg p.o.) administered orally, compared to (*rac*)-ketamine (10 mg/kg, i.p.) or GM-1020 (1.5 mg/kg, i.p.) on sucrose intake 24 h later. A one-way ANOVA revealed a significant main effect of treatment (F(6, 49) = 10.58, *p* < 0.0001). Asterisks indicate a significant difference from the vehicle + stress group using Dunnett’s post-hoc testing. All graphs show mean ± SEM (*n* ≥ 8/group). *p < 0.05, **p < 0.01, *** *p* < 0.001, ****p < 0.0001. ^#^ indicates significant difference between stressed and unstressed animals within the same treatment (*p* < 0.05).

GM-1020 (1-32 mg/kg, s.c.) produced dose-dependent reductions in immobility time in the FST 23.5 h post-dose (Fig. 2A). While all doses significantly reduced immobility time, the lowest dose achieving maximal efficacy was 3.2 mg/kg. GM-1020 produced reductions in immobility time comparable to desipramine.

### Chronic Mild Stress (CMS)

Rats exposed to CMS showed a robust anhedonic phenotype evidenced by a reduction in sucrose intake compared to control rats in weeks 1 and 2 of stress exposure; this reduction was maintained throughout the study in the vehicle group (Fig. 2C). In the first week of compound testing (week 3 of stress), administration of ketamine (10 mg/kg, i.p.) or GM-1020 (3 or 9 mg/kg, i.p.) reversed the deficit in sucrose intake in stressed rats measured 24 h after dosing. The two highest doses of GM-1020 (15 and 20 mg/kg, i.p.) caused substantial behavioral disruption in non-stressed animals, so animals from those two dose levels were re-randomized and assigned to receive either 0.75 or 1.5 mg/kg for the remaining trials. In weeks 4-7, ketamine and GM-1020 (1.5–9 mg/kg) reversed the effects of CMS on sucrose intake while the lowest dose of GM-1020 (0.75 mg/kg) was not effective. For weeks 8–10, stress continued but dosing ceased. The increase in sucrose intake caused by ketamine and GM-1020 was maintained in weeks 8–9, largely returning to stressed vehicle levels by week 10, indicating that the anti-anhedonic effects of both compounds were durable through 15 days after the final dose. The rapid and durable effects of (*rac*)-ketamine in CMS appear to model the well-documented rapid acting efficacy of (*rac*)-ketamine in patients with depression [28].

CMS-induced anxiety (decreased %time in the open arms) in the EPM (Fig. 2D) and memory impairment (decreased recognition index) in the NOR test (Fig. 2E) were also both reversed 48 h and 72 h, respectively, after administration of (*rac*)-ketamine or GM-1020. At the minimal efficacious dose (MED) of GM-1020 (1.5 mg/kg, i.p.) the peak plasma concentration was measured to be 151 ng/mL (Table S6).

In an additional experiment, the durability of a single administration of *(rac)-*ketamine or GM-1020 (1.5 or 9 mg/kg, i.p.), and oral administration of GM-1020 (1, 3.2, 10 mg/kg, p.o.), were assessed. The ability of a single dose of either GM-1020 or (r*ac*)*-*ketamine to restore sucrose intake was maintained for 14 days, with sucrose intake returning to stressed baseline levels 21 days after dosing (Fig. 2F). When administered orally, GM-1020 produced dose-dependent increases in sucrose intake, with 10 mg/kg restoring sucrose intake to unstressed levels 24 h after dosing (Fig. 2G). At the oral MED of GM-1020 (10 mg/kg), the peak plasma concentration was measured to be 257 ng/mL (Table S6).

### Spontaneous locomotor activity (sLMA)

**Fig. 3:**
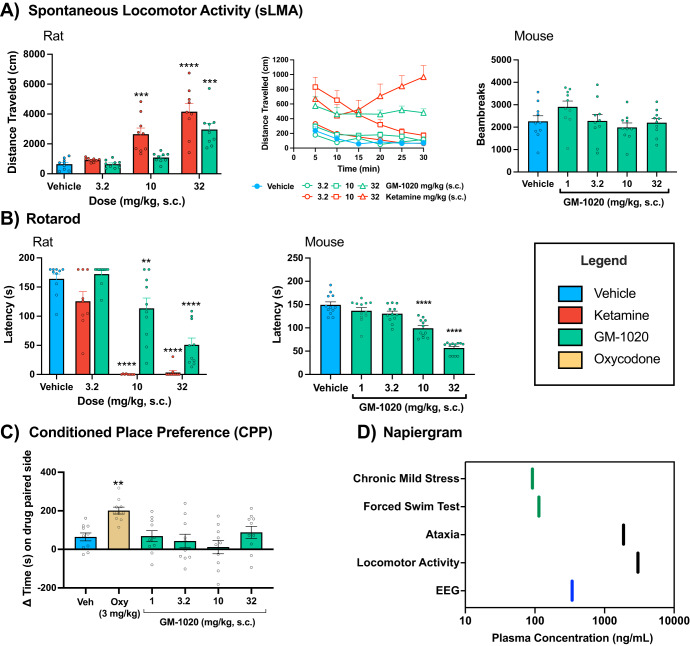
Motor effects of GM-1020 in rats and mice. **A** Spontaneous locomotor activity of rats (*n* = 9/group) dosed with vehicle, ketamine (3.2–32 mg/kg, s.c.) or GM-1020 (3.2–32 mg/kg, s.c.; left) and in mice (*n* = 10/group) dosed with GM-1020 (1–32 mg/kg, s.c.; right). Bar charts show total distance traveled over 0–30 min after dosing. Line graph (center) shows the time course of rat locomotor activity with data presented as 5 min bins, with rats dosed at time 0, and the given timepoint being the end of the 5 min bin (i.e. 5 min = 0–5 min). A one-way ANOVA revealed a significant main effect of treatment on the total distance traveled in the rats (F(6, 56) = 20.13, *p* < 0.0001). Dunnett’s post-hoc test showed significant differences between vehicle and ketamine at 10 mg/kg (*p* = 0.0001), and at 32 mg/kg (*p* < 0.0001), and between vehicle and GM-1020 at 32 mg/kg (*p* < 0.0001). For the mice, there was no significant effect of GM-1020 on spontaneous locomotor activity (F(4, 45) = 2.042, *p* = 0.1046). **B** Effects of vehicle, ketamine or GM-1020 (1–32 mg/kg, s.c.) on latency to fall off the rotarod 5 min after dosing in rats (left; *n* = 10/group) and mice (right; *n* = 12/group). For the rats, a one-way ANOVA revealed a significant main effect of treatment (F(6, 61) = 44.36, *p* < 0.0001). Post-hoc testing with Dunnett’s multiple comparison test revealed differences between vehicle treated animals and ketamine at 10 mg/kg (*p* < 0.0001) and 32 mg/kg (*p* < 0.0001), and between vehicle treated animals and GM-1020 at 10 mg/kg (*p* = 0.0070) and 32 mg/kg (*p* < 0.0001). Similarly in the mice, there was a significant main effect of treatment (F(4, 55) = 44.33, *p* < 0.0001) and Dunnett’s post-hoc test revealed significant differences between vehicle and 10 mg/kg and 32 mg/kg (*p’s* < 0.0001). **C** Effects of GM-1020 (1–32 mg/kg, s.c.) compared to oxycodone (3 mg/kg, s.c.) on mouse conditioned place preference (*n* = 10/group). The graph shows the change in time spent on the drug paired side after place conditioning. While the one-way ANOVA revealed a significant main effect of treatment (F(5, 54) = 5.091, *p* = 0.0007), Dunnett’s post-hoc test showed that only the group treated with oxycodone was significantly different from vehicle (*p* = 0.0065). **D** provides an overview of the plasma concentrations of GM-1020 associated with the pharmacodynamic effects of the drug. Green lines represent the plasma concentration of GM-1020 at the minimum efficacious doses in CMS and FST. Red lines indicate the plasma concentrations associated with a 50% reduction in latency to fall off the rotarod or a significant increase in spontaneous locomotor activity. The blue bar represents plasma concentrations of GM-1020 associated with decreases in EEG theta power and increases in gamma power in rats. Asterisks indicate significant differences compared to vehicle treated animals. *p < 0.05, **p < 0.01, ***p < 0.001, ****p < 0.0001. All graphs show mean ± SEM.

The effects of GM-1020 (3.2–32 mg/kg, s.c.) on sLMA in rats were compared to (*rac*)-ketamine (3.2–32 mg/kg, s.c.). Both compounds produced dose-dependent effects on sLMA with *(rac)*-ketamine producing robust increases at doses ≥10 mg/kg, and GM-1020 increasing activity only at 32 mg/kg (Fig. 3A; left). The exposure after GM-1020 (32 mg/kg, s.c.) in satellite animals was 3018 ng/mL, which was ~20-fold higher than the exposure at the MED in CMS, indicating a large separation between efficacious plasma exposures and those associated with motor side effects (Fig. 3D). In mice, GM-1020 (1-32 mg/kg, s.c.) did not affect locomotor activity (Fig. 3A; right).

### Rotarod

The ataxic effects of GM-1020 (3.2–32 mg/kg, s.c.) and (*rac*)-ketamine 3.2–32 mg/kg, s.c.) were explored in rats using the rotarod. Ketamine decreased the latency to fall off the rotarod at all doses tested, while GM-1020 produced decreases only at 10 and 32 mg/kg (Fig. 3B; left). The dose of GM-1020 resulting in a 50% reduction in latency to fall (ED_50_) was 17.4 mg/kg (interpolated plasma concentration = 1,876 ng/mL), whereas for (*rac*)-ketamine it was 3.4 mg/kg (interpolated plasma concentration = 217 ng/mL). Notably, the minimum dose of GM-1020 (10 mg/kg) to cause ataxia was associated with a plasma concentration of 1274 ng/mL in the satellite animals, ~8-fold higher than the concentrations at the MED in CMS (1.5 mg/kg, i.p.; 151 ng/mL; Fig. 3D).

Ataxic effects were also assessed in mice. Like in rats, GM-1020 did not produce ataxia at 1 or 3.2 mg/kg and had only a minor effect on the latency to fall at 10 mg/kg (Fig. 3B; right). The ED_50_ ataxic dose of GM-1020 in mice was 37.4 mg/kg, s.c.

### Conditioned place preference (CPP)

GM-1020 did not induce a CPP in mice at 1–32 mg/kg, s.c., while the animals treated with oxycodone (3 mg/kg, s.c.) as a positive control showed an increased time spent on the drug-paired side (Fig. 3C).

### Rat cortical EEG

**Fig. 4:**
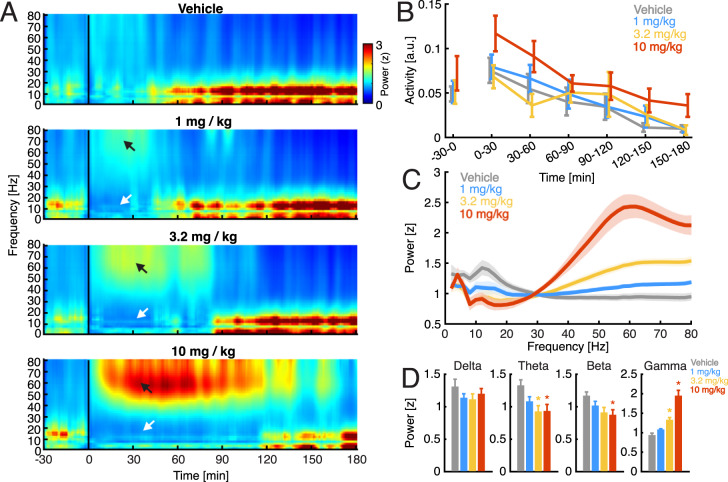
In vivo electrophysiology. **A** Spectrograms calculated for the time 30 min prior injection (*T* = 0) through 180 min after injection. Color represents z-scored EEG power, where z-score values are referenced to 30 min prior to injection. Black arrows point to an increase of broadband gamma EEG power. White arrows point to a decrease in low-frequency EEG power. **B** Average “activity” values in 30-min time windows before and after injection. The “activity” measure provided by DSI recording system reflects the movement of the animal across the home cage. Two-way ANOVA with repeated measures dose x time showed no difference between groups (dose: F(3, 168) = 1.78, *p* = 0.17; time: F(6, 168) = 6.45, *p* < 0.0001; dose × time: F(18, 168) = 0.64, *p* = 0.86). **C** Average spectra calculated at 60 min following injection. **D** Average values of EEG power calculated for 4 canonical EEG bands – delta (1–4 Hz; one-way ANOVA F(3, 31) = 1.03, *p* = 0.4), theta (5–12 Hz; one-way ANOVA F(3,31) = 4.63, *p* = 0.009, Tukey’s HSD test: Vehicle > 3.2 mg/kg and 10 mg/kg), beta (12-30 Hz; one-way ANOVA F(3, 31) = 3.59, *p* = 0.026, Tukey’s HSD test: Vehicle > 10 mg/kg) and gamma (30-80 Hz; one-way ANOVA F(3, 31) = 32.5, *p* < 0.0001, Tukey’s HSD test: Vehicle < 3.2 mg/kg < 10 mg/kg). Asterisks indicate a significant difference compared to vehicle treated animals. All data are the mean of *n* = 8/group and all error bars are SEM.

Effects of GM-1020 (1–10 mg/kg, s.c.) on the amplitude of cortical EEG oscillations were assessed for 30 min prior to and 180 min post injection (Fig. 4). GM-1020 produced a dose-dependent reduction in power in both theta (5–12 Hz) and beta (12–25 Hz) bands, while increasing power in the gamma (30–80 Hz) band. The effect on gamma was most pronounced in the 10 mg/kg group and peaked between 50–70 Hz. The effects lasted ~40 min in the 1 mg/kg group, ~90 min in the 3.2 mg/kg group, and ~120 min in the 10 mg/kg group (Fig. 4). The MED for decreasing theta and increasing gamma power was 3 mg/kg, which, based on subcutaneous rat PK, was associated with a peak plasma concentration of 342 ng/mL.

## Discussion

GM-1020 was developed to address two major shortcomings of ketamine: poor oral bioavailability and the narrow TI between antidepressant and ataxic/sedative doses. These features of ketamine require medical monitoring within a clinical setting. Preclinically, GM-1020 has greatly improved oral bioavailability and a wide margin between doses that produce antidepressant-like and ataxic effects. Translation of these findings to humans would represent a major improvement over traditional ketamine therapy for depression and enable greater patient access to a rapid-acting antidepressant.

A single dose of ketamine typically produces a reduction in depressive symptoms that begin immediately post-infusion and last for up to 7 days [1, 32]. This is already a major advantage over approved monoaminergic antidepressants, which can take weeks of dosing before producing improvements, and require patients to take the drug daily or multiple times per day [33, 34]. GM-1020 is a structural analog of ketamine with similar binding and electrophysiological effects in vitro. Like ketamine, GM-1020 binds to the MK-801 site within the ion channel of NMDAR and shows voltage-dependent inhibition, preferentially blocking activity in the hyperpolarized state. This voltage and state-dependent channel blockade is thought to be a key mechanism for the therapeutic effects of ketamine [35], and is a property shared by GM-1020.

In CMS, GM-1020 showed similar robust, rapid-acting, and durable antidepressant-like effects to ketamine. GM-1020 was effective at reversing the detrimental effects of stress on sucrose intake, anxiety, and memory with once weekly dosing. The rapid and durable effects of (*rac*)-ketamine in CMS appear to model the well documented rapid acting efficacy of (*rac*)-ketamine in patients with depression [28]. Because of the structural similarity and shared pharmacological target between GM-1020 and ketamine, we expect GM-1020 to also produce a rapid, robust antidepressant response in humans with intermittent dosing.

GM-1020 did not induce a CPP in mice, providing preliminary support for a low abuse potential. Ketamine has shown enantiomeric differences in assays of abuse liability with *(rac)*-ketamine and *S*-ketamine, but not *R*-ketamine inducing CPP in mice [11, 12]. Likewise, rats will self-administer *S*-ketamine but not *R*-ketamine [11]. As GM-1020 is more similar to *R*-ketamine than *S*-ketamine in stereochemistry, NMDAR potency, and off-target pharmacology, it is not surprising that it does not show abuse liability in this assay.

In the locomotor and rotarod assays, rats were less affected after administration of GM-1020 compared to ketamine. Importantly, the motor impairment seen in these assays following GM-1020 occurred at doses/exposures far higher than those that were efficacious in CMS. For GM-1020, 1.5 mg/kg (i.p.) was fully efficacious in CMS and no impairment in rotarod performance was seen at 3.2 mg/kg (s.c.), and only mild effects at 10 mg/kg (ED_50_ = 17.4 mg/kg, s.c.). Using plasma concentrations to compare across studies which used different routes of administration (ROA), the peak plasma concentration of GM-1020 associated with the calculated ED_50_ for latency to fall off the rotarod was interpolated as 1,876 ng/mL. This is ~12-fold higher than the C_max_ measured after administration of GM-1020 at 1.5 mg/kg (i.p.; 151 ng/mL), the MED in CMS, indicating a large separation between antidepressant-like efficacy and motor side effects.

In contrast, while ketamine 10 mg/kg (i.p.) was fully efficacious in CMS, lower doses were not evaluated so this has not been confirmed as the MED. However, in the second CMS study in WKY rats, the effect of ketamine (10 mg/kg, i.p.) on sucrose intake failed to reach statistical significance, suggesting that this dose in Wistar Han rats is near the MED and at most, is ~½ a log unit above the MED. A dose of 10 mg/kg (s.c.) ketamine completely disrupted animals’ ability to stay on the rotarod (ED_50_ = 3.4 mg/kg, s.c). Using plasma concentrations to compare across studies using different ROAs, the peak concentration of ketamine associated with the ED_50_ for latency to fall off the rotarod was interpolated as 172 ng/mL. This is <2-fold higher than the peak exposure after administration of ketamine in CMS (10 mg/kg, i.p.; 125 ng/mL or 131 ng/mL [36]). Even allowing that the true MED of ketamine could be 3.2 mg/kg (i.p.; ~½ log unit lower than the lowest dose tested, as discussed above), this would achieve a peak exposure of ~40 ng/mL and would result in a ~4-fold separation between antidepressant-like efficacy and motor side effects, substantially less than that of GM-1020.

It is not clear from these data why GM-1020 has a greater therapeutic window than ketamine, and more mechanistic studies are needed to understand the improved tolerability of this molecule. One hypothesis is that while dissociative and sedative/motor side effects are clearly related to peak drug exposures (‘*C*_max_-driven’) it is feasible/likely that AUC exposure contributes to efficacy observed ≥24 h after dosing. In rats the pharmacokinetic profile of GM-1020 (administered i.p.; *C*_max_ 151 ng/mL, AUC_0–24h_ 168 h ng/mL) results in a greater AUC_0–24h_ to *C*_max_ exposure ratio compared to ketamine (i.p.; *C*_max_ 402 ng/mL, AUC_0–24h_ 179 h ng/mL). To achieve an equivalent AUC exposure to GM-1020, ketamine needs to be dosed to a much greater C_max_, resulting in a greater side effect burden.

However, this hypothesis does not explain the data with *R*-ketamine, which has similar NMDAR affinity to GM-1020, and is reportedly less dissociative at antidepressant doses [10]. Preclinically, *R*-ketamine has been shown to produce a different behavioral phenotype than racemic or *S*-ketamine [12, 13]. At the receptor level, it is difficult to explain such a right shift or ceiling on side effects without a corresponding right shift or ceiling on antidepressant effects, thus more research is needed. In a recent study, Mathai et al. found there was no relationship between acute dissociative effects and the antidepressant response to esketamine [37]. Likewise, in a review by Ballard and Zarate, the authors argue that the current body of literature does not support the hypothesis that the dissociative effects of ketamine are required for an antidepressant response [38].

It remains to be seen whether GM-1020 will have antidepressant efficacy in humans at non-dissociative doses. Demonstrating that GM-1020 can modulate EEG power in healthy subjects, indicative of NMDAR target engagement, without causing dissociation or sedation, would provide insight into the likelihood of an improved TI and is being investigated in an ongoing Phase I clinical trial.

While the effects of NMDAR antagonists on ataxia show good translation between rodents and humans, the greater challenge is in predicting antidepressant efficacy in humans from rodent preclinical data. Therefore, EEG was chosen as a quantitative translational biomarker of target engagement. Ketamine reduces power in low frequency bands and increases power in the gamma band both preclinically and clinically [29, 39–41]. The reduction of low frequency activity and increase in gamma seen in the EEG experiment with GM-1020 demonstrate clear target engagement, consistent with NMDAR blockade. While the exact mechanism of EEG gamma power increase following NMDAR blockade is still unclear, the likely mechanism involves disinhibition of pyramidal cells which in turn increases the drive on highly connected local interneurons therefore temporarily synchronizing reciprocal excitatory-inhibitory interactions within neural networks resulting in increased amplitude of observed gamma oscillations [42, 43]. A dose of 3 mg/kg (s.c.), which achieves similar exposure to the minimum efficacious dose in CMS (1.5 mg/kg, i.p.), was sufficient to affect this biomarker of NMDAR blockade, which demonstrates this dose of GM-1020 is producing significant engagement of NMDAR in vivo. In a recently published study evaluating the dose response of ketamine in rat EEG, the minimum dose that produced a significant increase in gamma activity in SD rats was 10 mg/kg (s.c.) [29]. The same study did not detect an effect of ketamine (up to 10 mg/kg) on the lower frequency bands while the animals were awake, except for a small increase in beta power at 10 mg/kg in WKY rats [29], which contrasts with the decrease observed with GM-1020.

The data presented demonstrate that GM-1020 and ketamine share a common mechanism as NMDAR channel blockers and that GM-1020 displays similar antidepressant-like efficacy with an associated quantitative signal of target engagement. A limitation of the current study is the exclusive use of male rodents. While it cannot be ruled out that GM-1020 could have different dose-dependent effects in female rodents, a recent meta-analysis of clinical trials with esketamine concluded there were no sex differences in either safety or efficacy [44]. Thus, GM-1020 is well positioned to exhibit rapid-acting antidepressant effects in humans. Both the physiochemical and pharmacokinetic properties of GM-1020 indicate that it is well-suited for development as an oral medication. Although GM-1020 was administered parenterally in most behavioral studies, exposures were achieved that can be equated to oral doses, so there is no reason to expect differences in preclinical efficacy based on the ROA. Additionally, a single oral dose of GM-1020 was efficacious in CMS, further supporting development as an oral medication. The predicted ~60% oral bioavailability of GM-1020 in humans is greatly improved compared to ~17-24% reported for ketamine [45].

GM-1020 is a significant advancement in the field of rapid-acting antidepressants. An orally bioavailable rapid-acting antidepressant will allow for greater patient access, negating the need for infusion clinics or requirement of drug-delivery devices to administer the medication. This alone is a significant improvement over the current practice of ketamine therapy. If GM-1020 has reduced dissociative/sedative effects in humans at an efficacious dose, as predicted from the preclinical data, it may allow for at-home dosing, without the need for clinical monitoring, further expanding patient access. Finally, if confirmed clinically, a less-dissociative NMDAR antagonist would represent a significant advancement in our understanding of the relationship between NMDAR function and behavior, demonstrating it is possible to separate therapeutic efficacy from dissociative side effects.

### Supplementary information

Supplemental Material
