# Supplementary material for: GM-1020: a novel, orally bioavailable NMDA receptor antagonist with rapid and robust antidepressant-like effects at well-tolerated doses in rodents
Source: Neuropsychopharmacology. 2024 Jan 4;49(6):905–14. doi: 10.1038/s41386-023-01783-1 (PMC11039472; doi:10.1038/s41386-023-01783-1)
Supplement: Supplementary file 1 — Supplemental Material [file 41386_2023_1783_MOESM1_ESM.docx]

**Supplementary Materials and Methods**

**Materials**

GM-1020 ((R)-2-(4-fluorophenyl)-2-(methylamino)cyclohexan-1-one) was synthesized by WuXi AppTech (Shanghai, China). *R-* and *S-*Ketamine were purchased from Tocris Bioscience (Bristol, UK) and Cayman Chemical (Ann Arbor, MI). (*rac*)-Ketamine (Ketalar) was from Pfizer (Zurich, Switzerland) while L-Glutamate and L-Glycine, antibiotics and (2*R*)-amino-5-phosphonovaleric acid (D-AP5) were purchased from Sigma-Aldrich (St. Louis, Missouri). Vetoquinol ((*rac*)-ketamine; Biowet, Gorzow Wielkopolski, Poland) was used for CMS studies. Compounds administered *in vivo* were dissolved in sterile saline.

**In Vitro Pharmacology**

Competitive radioligand binding experiments at the NMDA receptor with [^3^H]MK-801 were carried out by Eurofins Panlabs (Taipei, Taiwan) under the conditions outlined in Table S1, while MOR binding was carried out by Wuxi Biologics (China) with the conditions in Table S2, and functional activity was assessed via FLIPR. The Cerep87 SafetyScreen panel was carried out by Eurofins Cerep (Lebois L’eveque, France) on GM-1020 at 10 μM to determine any interactions with receptors, transporters or enzymes that have known safety liabilities.

**NMDAR Patch clamp studies**

In vitro electrophysiology studies in oocytes were conducted by Ecocyte Bioscience (Dortmund, Germany). Oocytes were harvested from adult *Xenopus laevis* and incubated in a 96-well plate for 2-4 days. Expression of hNMDA receptor subunits was achieved via injection of 100 ng/µl cDNA per subunit using the Roboocyte automated injection system (Multichannel Systems, Germany). For recordings, 96-well plates were transferred to the Roboocyte system, and each oocyte was pre-injected with 4 mM sodium BAPTA calcium chelator before recording. Membrane potentials were clamped to a holding potential of -70 mV and induced currents after compound application were recorded at a sampling frequency of 200 Hz at room temperature. Currents were monitored by the Roboocyte software and stored for offline analysis. For each compound and solution N=5-10 oocytes were recorded and used in the analysis. The mean of the glutamate- and glycine-induced steady-state currents at the end of each application (last 5 s of 60 s) of all tested oocytes were imported into Graphpad Prism 7 and analyzed to determine IC50 values. The unblocking kinetics were analyzed as previously described [21]. Curve fitting was done with 𝑌=𝐵𝑜𝑡𝑡𝑜𝑚+(𝑇𝑜𝑝−𝐵𝑜𝑡𝑡𝑜𝑚)(1+10𝐿𝑜𝑔𝐸𝐶50−𝑋)⁄, with “Bottom” and “Top” as minimal and maximal response.

In vitro electrophysiology studies in HEK cells were conducted by B’Sys (Witterswill, Switzerland) with QPatch 16X or QPatch HTX (Sophion Bioscience, Ballerup, Denmark). HEK293 stably transfected with tetracycline inducible GRIN1/2A subunits (NR1: NP_015566.1 NR2A: AAB49993.1) were maintained in HAM/F-12 (with L-glutamine) supplemented with 10% fetal bovine serum 1.0% Penicillin/Streptomycin solution and 100 μM AP5 plus selection antibiotics: hygromycin 500 μg/mL, puromycin 5.0 μg/mL, zeocin 100 μg/mL. Tetracycline 2.5 μg/mL was added 24 h before the start of electrophysiology experiments. Cells were washed twice with PBS after detachment and transferred as suspension in PBS to the QPatch 16X or QPatch HTX system followed by two further washing steps with bath solution. The 1x bath solution was prepared every week by diluting 10x bath solution without glucose and 100x glucose solution with water (ELGA PURELAB Flex 03). Stock solutions were stored at 4°C (10x bath solution) or -20°C (100x glucose solution). When in use, the 1x bath solution was kept at room temperature (RT). 1x Bath solution: NaCl 137 mM (Sigma Aldrich, order number: 71380 no Mg^2+^ contamination), KCl 4 mM, CaCl_2_ 1.8 mM (2.8 mM after giga seal formation), MgCl_2_ 1 mM, HEPES 10 mM, D-Glucose 10 mM pH (NaOH) 7.4. 1x Bath solution without Mg^2+^: NaCl 145 mM, KCl 4 mM, CaCl_2_ 0.2 mM, HEPES 10 mM, D-Glucose 25 mM pH (NaOH) 7.4 Test items were dissolved or diluted in bath solution containing glutamate (100 μM) and glycine (10 μM) to achieve a concentration of 300 μM. Lower concentrations were prepared by serial dilution. Fresh solutions of test items were prepared every day and maintained at RT when in use. The 1x intracellular (pipette) solution included the following components: KF 120 mM, KCl 10 mM, MgCl_2_ 1 mM, Mg-ATP 5 mM, HEPES 10 mM, EGTA 10 mM pH (KOH) 7.2. The pipette solution was aliquoted and stored at -20°C. Only one concentration of compound was tested per cell and at least 5 cells per experimental group.

**Application protocol current inhibition and off-rate**

Outward currents were measured upon depolarization of the cell membrane to +60 mV for 4 s from a holding potential of −80 mV. Recording started and ended 500 ms before or after the voltage change to +60 mV or to -80 mV. Test solutions were applied 500 ms after voltage change to +60 mV. The waiting time between two voltage pulses was 40 s.

1 Bath solution

2 Bath solution + Glutamate / Glycine

3 Bath solution + Glutamate / Glycine + test item (determination of current inhibition)

4 Bath solution + Glutamate / Glycine (determination of off rate)

Increase of the current amplitude after washing off the test item was fit with an exponential function and the off rate and half-life time were determined (n≥5). Additionally, the current inhibition during wash-in of the test items was analyzed. The current amplitude in the presence of test item was normalized to the current amplitude before application of test item. These values were fit with a logistic equation (E_max_: 100%) to determine the IC_50_ value and Hill coefficient at a potential of +60 mV.

The application protocols for IC50 determination and voltage dependence can be found in Tables S3 and S4, respectively.

**Microsomal metabolism**

The microsomal metabolism of GM-1020 (1 μM final conc - 1% w/v DMSO) was evaluated at 37°C in presence of 1 mM NADPН using liver microsomal protein (0.5 mg/ mL) from male Sprague Dawley rats (R-1000), male CD-1 mice (M-1000), male Gottingen minipigs (Z6000), male beagle dogs (D-1000) male cynomolgus monkey (P2000) and human liver pooled from female and male donors (H0610) from Xenotech (Kansas City, KS). All studies were carried out by WuXi (Shanghai, China) [1].

Microsomal incubations were carried out in multi-well plates. Liver microsomal incubation medium consisted of PBS (100 mM, pH 7.4), MgCl_2_ (1 mM), and NADPН (1 mM), with 0.50 mg of liver microsomal protein per mL. Control incubations were performed by replacing the NADPH-cofactor system with PBS. Test compounds (1 μM, final solvent concentration 1.0%) were incubated with microsomes at 37 °C with constant shaking. Six time points over 60 min were analyzed, with 60 μL aliquots of the reaction mixture being drawn at each time point. The reaction aliquots were stopped by adding 180 μL of cold (4°C) acetonitrile containing 200 ng/mL tolbutamide and 200 ng/mL labetalol as internal standards (IS), followed by shaking for 10 minutes, and then protein sedimentation by centrifugation at 4,000 rpm for 20 minutes at 4 °C. Supernatant samples (80 μL) were diluted with water (240 μL) and analyzed for parent compound remaining using a fit-for-purpose liquid chromatography-tandem mass spectrometry (LC-MS/MS) method.

**Pharmacokinetic Studies**

Compounds were administered to rats and mice fasted for 4h before dosing and in larger mammals fasted overnight. Pharmacokinetic studies in adult male SD rats and C57BL/6 mice were conducted at Sai Life Sciences (Pune, India), and in bama minipigs, cynomolgus monkeys, and beagle dogs at ChemPartner (Shanghai, China). All procedures were in accordance with the relevant State guidelines and with prior approval of the institutional animal ethics committee.

Blood samples (8 x ~60 µL samples/subject; n=4 subjects) were collected from rodents (under light isoflurane anesthesia (Surgivet^®^) from the retro orbital plexus (0.083, 0.25, 0.5, 1, 2, 4, 8, 24 hours). Blood samples (10 samples/subject; n=3 subjects) from large mammals (pre-dose, 0.083, 0.25, 0.5, 1, 2, 4, 8, 12, and 24 hours) were collected under manual restraint from the cephalic vein. Plasma was stored at -80ºC until bioanalysis by LC-MS/MS method. For studies with brain collection, immediately following blood collection, the abdominal vena-cava was cut open under deep anesthesia and the whole body was perfused from the heart using 10 mL of normal saline. Brains were collected from four rats at each specified time point. After isolation, brain tissue samples were rinsed three times in ice cold normal saline and dried on blotting paper. Tissue samples were homogenized using ice-cold phosphate buffered saline (pH 7.4). Total homogenate volume was three times the tissue weight. All homogenates were stored at -70 ± 10 ºC until bioanalysis. Pharmacokinetic parameters were estimated using the non-compartmental analysis tool of Phoenix^®^ WinNonlin software (8.0) (Certara, Princeton, NJ).

**Forced Swim Test studies**

Forced Swim Test (FST) experiments were performed by Adgyl Lifesciences (Bengaluru, India) using groups of n=10-20, 8 to 10-week-old male Sprague-Dawley (SD) rats (Hylasco Biotechnology, India). Rats were housed 2 per cage with a 12 h light/12 h dark cycle, and food and water provided *ad libitum*. Animals were distributed to different groups based on body weight and groups were randomly assigned to treatment conditions. Procedures involving animals were performed according to the guidelines approved by the Institutional Animal Ethics Committee of Adgyl Lifesciences and according to the National Institutes of Health Guide for Care and Use of Laboratory Animals.

Animals first had a training swim where they were placed in individual glass cylinders (46 cm x 20 cm) containing 23-25º C water at a depth of 30 cm for 15 mins. Animals were dried and warmed after removal from the water.

Thirty minutes after the pre-swim (and 23.5 h prior to the test swim), animals were treated once with GM-1020 (1 – 32 mg/kg, s.c.) or vehicle. Another group of animals was treated with desipramine (20 mg/kg, s.c.) three times (23.5h, 5h, 1h prior to test swim) as a positive control. The test swim was conducted 24 hours after the pre-swim using the same apparatus. During this test swim, immobility time was scored for 5 min by an observer blinded to the treatment condition. Data were analyzed using GraphPad Prism 9.

**Chronic Mild Stress**

The chronic mild stress paradigm was performed as previously been described [2]. Adult male Wistar Han rats (270-375 g at baseline) were subjected to stressors for a total of 10 weeks. After two weeks of CMS, animals were dosed once weekly with GM-1020 (0.75, 1.5, 3, 9 mg/kg; i.p.), ketamine (10 mg/kg; i.p.), or vehicle (i.p.), for 5 weeks. Initial doses of 15 and 22.5 mg/kg were trialed and discontinued after one dose due to behavioral disruptions and those animals were re-randomized to the 0.75 and 1 mg/kg groups for subsequent weeks. Stress exposure continued for an additional 3 weeks after the final dose. Anhedonia was assessed weekly by measuring sucrose intake (24h after dosing). Anxiety and memory were assessed using the elevated plus maze (EPM) and novel object recognition (NOR) tests, which were performed 48 and 72 h after the first administration of test compounds, respectively. In a second CMS study in male WKY rats (230-340 g at baseline), the same procedures were followed as described above, however NOR and EPM testing was not performed.

For the sucrose intake analysis, a Mixed Model with Repeated Measures statistical test was used due to missing data at one timepoint; other experiments were analyzed with one or two-way ANOVA using GraphPad Prism 9. Procedures used in this study conformed to the rules and principles of the 86/609/EEC Directive and were approved by the Local Bioethical Committee at the Institute of Pharmacology, Polish Academy of Sciences, Krakow, Poland.

**Spontaneous Locomotor Activity: Rats**

Locomotor activity assessment was performed by Adgyl Lifesciences (Bengaluru, India) using groups of n=9, 8-10 week old male Sprague-Dawley (SD) rats (Hylasco Biotechnology, India). Rats were housed 2 per cage with a 12h light/12h dark cycle, and food and water provided *ad libitum*. Animals were distributed to different groups based on body weight and groups were randomly assigned to treatment conditions. Procedures involving animals were performed according to the guidelines approved by the Institutional Animal Ethics Committee of Adgyl Lifesciences and according to the National Institutes of Health Guide for Care and Use of Laboratory Animals.

Thirty minutes prior to testing, rats were acclimated to the testing room. After acclimation to the room, animals were individually placed in photobeam activity chambers (Columbus Instruments, USA) where the animals underwent a 30-min acclimation period. Rats were then removed from the chamber and injected with GM-1020 (3.2-32 mg/kg, s.c.), *(rac)*-ketamine (3.2-32 mg/kg, s.c.) or vehicle. Immediately post-injection, animals were individually placed in the previously used activity chambers and activity measured for 30 minutes. Activity was expressed as the total distance traveled (cm). Data were analyzed using a one-way ANOVA with GraphPad Prism 9.

**Motor Coordination (Rotarod): Rats**

Motor coordination studies were performed by the Neuropharmacology Core at The University of Mississippi School of Pharmacy using groups of n=10 (8-10 week old) male SD rats (Envigo, Indianapolis, IN) housed 2 to 4 per cage under a 12h/12h light /dark cycle and with ad libitum access to food and water. Procedures were performed according to the guidelines approved by the Institutional Animal Care and Use Committee of the University of Mississippi where the experiments were carried out and according to the National Institutes of Health Guide for Care and Use of Laboratory Animals. Animals were tested during the light phase of their light cycle. Animals were distributed to different groups based on body weight and groups were randomly assigned to treatment conditions.

Rats were first trained to balance on an immobile rotarod (San Diego Instruments, San Diego, CA, USA) (2.75” diameter, 48” fall height) for 30 s. Animals were then assessed on the rotating rotarod across three fixed-speed trials (30 s max latency at 10 RPM), two fixed speed trials (180 s max latency at 10 RPM), and two accelerated speed trials (180 s max latency at 0-20 RPM). After each trial the latency to fall from the rotarod was recorded. The last of these trials was used as a baseline measure of rotarod performance. Twenty-four hours later, rats were administered GM-1020 (3.2-32 mg/kg, s.c.), *(rac)-*ketamine (3.2-32 mg/kg, s.c.) or vehicle and assessed in an accelerated speed trial (180 s max latency at 0-20 RPM) at 5 minutes post-dose. Data were analyzed using a one-way ANOVA with GraphPad Prism 9.

**Spontaneous Locomotor Activity: Mice**

Locomotor assessments were additionally conducted in mice by the Neuropharmacology Core at The University of Mississippi School of Pharmacy using groups of n=10, 8–10-week-old male C57BL/6 mice (Envigo, Indianapolis, IN). Mice were housed 2 per cage with a 12 h light/12 h dark cycle, and food and water provided *ad libitum*. Animals were distributed to different groups based on body weight and groups were randomly assigned to treatment conditions. Procedures were performed according to the guidelines approved by the Institutional Animal Care and Use Committee of the University of Mississippi where the experiments were carried out and according to the National Institutes of Health Guide for Care and Use of Laboratory Animals.

Thirty minutes prior to testing, mice were acclimated to the testing room. After acclimation to the room, animals were individually placed in photobeam activity chambers (Columbus Instruments, USA) where the animals underwent a 30-minute acclimation period. Mice were then removed from the chamber and injected with test compound. Immediately post-injection, animals were individually placed in the previously used activity chambers and locomotor activity was measured for 30 minutes. Activity is expressed as the total number of beambreaks. Data were analyzed using a one-way ANOVA with GraphPad Prism 9.

**Motor Coordination (rotarod): Mice**

Mouse motor coordination studies were performed by Adgyl Lifesciences (Bengaluru, India) using groups of n=12 8–10-week-old male C57BL/6 mice (Hylasco Biotechnology, India) housed 2 per cage under a 12 h/12 h light/dark cycle with *ad libitum* access to food and water. Procedures involving animals were performed according to the guidelines approved by the Institutional Animal Ethics Committee of Adgyl Lifesciences and according to the National Institutes of Health Guide for Care and Use of Laboratory Animals. Mice were tested during the light phase of their light cycle. Animals were distributed to different groups based on body weight and groups were randomly assigned to treatment conditions.

Mice were trained to balance on a rotarod (San Diego Instruments, San Diego, CA) (1.25” diameter, 18” fall height) rotating at 4 RPM for 3 mins, three times per day over two days, followed by two days at 15 RPM for 3 mins. Animals that fell off before 80s during any training session were excluded from the study. Twenty-four hours later, mice were administered GM-1020 or vehicle and assessed in an accelerated speed trial (300 s max latency at 0-40 RPM) at 5 minutes post-dose. Data were analyzed using a one-way ANOVA with GraphPad Prism 9.

**Conditioned Place Preference (CPP)**

CPP studies were carried out in groups of n=10 male 8–10-week-old C57BL6/J mice (Envigo, Indianapolis, IN) by the Neuropharmacology Core at The University of Mississippi School of Pharmacy. Animals were conditioned with GM-1020 (1-32 mg/kg; s.c.), oxycodone (3 mg/kg; s.c.) as a positive control, or vehicle. Procedures were performed according to the guidelines approved by the Institutional Animal Care and Use Committee of the University of Mississippi where the experiments were carried out and according to the National Institutes of Health Guide for Care and Use of Laboratory Animals.

The procedure consists of three phases and includes 1) an acclimation day followed by a baseline preference test the following day (15 min) to determine conditioning compartment preference, 2) four sets of S+/S- conditioning (drug/no drug), and 3) a final preference test following conditioning trials. PHASE 1: The acclimation-baseline preference trial consists of placing the animal in the grey center chamber, opening the guillotine door after 5 min, allowing the animal to explore the black versus white conditioning compartments (and gray chamber) during a 15 min period. The results of the baseline preference trial identify initial compartment preference and permit the assignment of S+ (drug) treatment to the non-preferred chamber and S- (vehicle) to the preferred chamber. This was done in counter-balanced design across the experimental conditions. PHASE 2: Mice receive four sets of S+/S- conditioning trials on alternating days where they are confined to the assigned chamber for 45 mins after drug or vehicle injection. PHASE 3: Final preference test involves measurement of the relative preference for the S+/S- compartments. This preference trial was conducted in a drug-free state the day following the fourth conditioning trial. Each animal was placed in the grey center chamber for 5 min followed by opening of the guillotine door allowing access to explore the black versus white conditioning compartments (and gray chamber) during a 15 min period. A conditioning score was calculated by subtracting the time spent in the drug paired compartment pre versus post conditioning.

**EEG Studies**

A cohort of 8 rats (Charles River, UK) was used to assess the effects of GM-1020 (1-10 mg/kg; s.c.) and vehicle on cortical EEG at Transpharmation, UK using methodology previously described [3]. At least 1 week after surgery, rats received each treatment in a pseudo randomised cross-over fashion with a minimum period of 7 days between doses. Baseline recordings were conducted for 1 h prior to dosing and EEG was recorded for 23 h post-dose. Effects of treatment and time were assessed with 2-way repeated-measures ANOVA (R version 3.6.3). Post-hoc analysis of interaction contrasts was performed to compare each treatment with the vehicle with Dunnett’s correction applied. Instantaneous EEG power was computed using custom scripts written in Matlab (Natick, MA). EEG signals were convolved with a group of complex Morlet wavelets [4] in the frequency range of 2-80 Hz with step of 2 Hz and scaling factor f_0_/σ_f_ = 8 followed by squaring the result. Next, frequency-specific EEG powers were z-score normalized by dividing the power values by a square of standard deviation of filtered signal obtained from baseline 30-minute pre-injection period. Next, normalized power values were either averaged in 10-second-long windows to obtain spectrograms (Fig. 4A) or averaged across 60-minute post-injection period to obtain power spectra (Fig. 4C). Normalized power values were further averaged across canonical EEG bands delta (δ: 1-4 Hz), theta (θ: 5-12Hz), beta (β: 12-30 Hz), and gamma (γ: 30-80 Hz) to obtain band-specific spectral averages (Fig. 4D). Statistical differences were evaluated using one-way ANOVA followed by post hoc Dunnett’s test for differences from vehicle.

**Table S1.** NMDAR radioligand binding experimental parameters.

| **Receptor Source** | Wistar rat brain (minus cerebellum) |
| --- | --- |
| **Vehicle** | 1.0% DMSO |
| **Incubation Time** | 3 h |
| **Incubation Temperature** | 25 °C |
| **Incubation Buffer** | 5 mM Tris-HCl, pH 7.4 |
| **Ligand** | 5.0 nM [^3^H]MK-801 |
| **Non-Specific Ligand** | 10.0 µM (+)-MK-801 |
| **Specific Binding** | 90%* |
| **K_d_** | 12.0 nM* |
| **B_max_** | 1.30 pmol/mg protein* |
| *historical values |  |

**Table S2:** MOR radioligand binding studies

| **Receptor Source** | hMOR-HEK293 cellular membranes |
| --- | --- |
| **Vehicle** | 0.5% DMSO |
| **Incubation Time** | 1 h |
| **Temperature** | 25 °C |
| **Incubation Buffer** | 50 mM Tris-HCl, 5 mM MgCl_2_ pH 7.4 |
| **Ligand** | 0.5 nM [^3^H]-DAMGO |
| **Non-Specific Ligand** | 0.1 µM DAMGO |
| **Specific Binding** | 88% |
| **K_d_** | 0.280 nM |
| **B_max_** | - 1. pmol/mg |

**Table S3. Application protocol IC_50_ studies**

| Application No. | Duration | With pre-wash of test item |
| --- | --- | --- |
| 1 | 30 s | Bath solution |
| 2 | 10 s | Glutamate + Glycine |
| 3 | 30 s | Bath solution |
| 4 | 10 s | Glutamate + Glycine |
| 5 | 30 s | Bath solution |
| 6 | 10 s | Glutamate + Glycine |
| 7 | 30 s | Test item 0.4 μM |
| 8 | 10 s | Test item 0.4 μM + Glutamate + Glycine |
| 9 | 30 s | Test item 1.2 μM |
| 10 | 10 s | Test item 1.2 μM + Glutamate + Glycine |
| 11 | 30 s | Test item 4.0 μM |
| 12 | 10 s | Test item 4.0 μM + Glutamate + Glycine |
| 13 | 30 s | Test item 12 μM |
| 14 | 10 s | Test item 12 μM + Glutamate + Glycine |
| 15 | 30 s | Test item 40 μM |
| 16 | 10 s | Test item 40 μM + Glutamate + Glycine |

**Table S4. Application protocol state dependence studies**

| **Application No.** | **Duration** | **Without pre-wash of test item** | **With pre-wash of test item** |
| --- | --- | --- | --- |
| 1 | 30 s | Bath solution | Bath solution |
| 2 | 10 s | Glutamate + Glycine | Glutamate + Glycine |
| 3 | 30 s | Bath solution | Bath solution |
| 4 | 10 s | Glutamate + Glycine | Glutamate + Glycine |
| 5 | 30 s | Bath solution | Bath solution |
| 6 | 10 s | Glutamate + Glycine | Glutamate + Glycine |
| 7 | 30 s | Bath solution | Test Item |
| 8 | 10 s | Test item + Glutamate + Glycine | Test item + Glutamate + Glycine |
| 9 | 30 s | Bath solution | Test Item |
| 10 | 10 s | Test item + Glutamate + Glycine | Test item + Glutamate + Glycine |

**Supplementary Data**

**Table S5. Cerep Binding Panel**

| **Receptor** | **% Inhibition at 10 µM** |
| --- | --- |
| Adenosine_1_ | 1.6 |
| Adenosine_2A_ | -15.8 |
| α_1A_ adrenergic | -0.9 |
| α_1B_ adrenergic | -2.3 |
| α_1D_ adrenergic | -15.2 |
| α_2A_ adrenergic | 13.4 |
| α_2B_ adrenergic | -5.8 |
| β_1_ adrenergic | 7.8 |
| β_2_ adrenergic | -1.2 |
| AT_1_ | 1.7 |
| BZD (central) | 11.5 |
| Cl- channel (GABA-gated) (TBOB site) | -12.8 |
| B_2_ | -7.6 |
| CB_1_ | -16.5 |
| CB_2_ | -1.6 |
| CCK_1_ | -17.0 |
| CCK_2_ | 0.8 |
| D_1_ | 6.0 |
| D_2S_ | 14.1 |
| D_2L_ | -3.1 |
| ET_A_ | 3.2 |
| GABA_A_ (α_1_β_2_γ_2_) | -0.6 |
| GABA_B(1b)_ | 3.0 |
| **AMPA** | **18.3** |
| Kainate | 2.4 |
| NMDA | 9.6 |
| Glycine (strychnine-sensitive) | -18.6 |
| Glycine (strychnine-insensitive) | -12.0 |
| CXCR2 (IL-8B) | -7.8 |
| CCR1 | -6.8 |
| H_1_ | 0.6 |
| H_2_ | -17.4 |
| CysLT_1_ (LTD_4_) | 1.9 |
| MC_1_ | 5.2 |
| **MC_4_** | **22.6** |
| MAO-A | 6.9 |
| M_1_ | 8.0 |
| M_2_ | 3.6 |
| M_3_ | 1.4 |
| M_4_ | 13.7 |
| NK_1_ | -13.9 |
| Y_1_ | 6.8 |
| nA**ChR (neuronal) α4β2** | **17.8** |
| NAChR (muscle-type) | 4.0 |
| δ-opioid | -5.2 |
| κ-opioid | 11.5 |
| µ-opioid | 10.5 |
| PPARγ | 6.7 |
| **PCP** | **68.3** |
| RARα | -11.1 |
| 5-HT_1A_ | -12.3 |
| **5-HT_1B_** | **29.1** |
| 5-HT_2A_ | -6.0 |
| 5-HT_2B_ | 2.8 |
| 5-HT_2C_ | 8.9 |
| 5-HT_3_ | -4.7 |
| GR | -17.7 |
| ER_α_ | -22.1 |
| PR | -13.7 |
| AR | -12.1 |
| V_1a_ | 3.2 |
| L-type calcium channel (dihydropyridine site) | -13.2 |
| L-type calcium channel (diltiazam site) | -7.5 |
| L-type calcium channel (verapamil site) | -6.4 |
| N-type calcium channel | -5.6 |
| hERG ([^3^H]dofetilide) | 0.4 |
| K_v_ | 4.5 |
| **Sodium channel (site 2)** | **17.8** |
| Adenosine transporter | -0.1 |
| Norepinephrine transporter | -10.9 |
| Dopamine transporter | 15.6 |
| GABA transporter | -3.5 |
| **5-HT transporter** | **39.8** |

**Table S6. GM-1020 Rat Pharmacokinetics (SC, IP, PO)**

| Species and Number | Route | Dose | T_max_  (h) | C_max_  (ng/ml) | AUC _(0-t)_ (h*ng/ml) | T_1/2_  (h) |
| --- | --- | --- | --- | --- | --- | --- |
| Rat. SD, 4M | SC | 3.0 mg/kg | 0.5 | 341.6 | 603.8 | 0.98 |
|  | IP | 1.5 mg/kg | 0.25 | 151.1 | 167.4 | 0.72 |
|  | PO | 10 mg/kg | 0.69 | 256.48 | 754.0 | 1.70 |

**References**

1. Turfus SC, Parkin MC, Cowan DA, Halket JM, Smith NW, Braithwaite RA, et al. Use of Human Microsomes and Deuterated Substrates: An Alternative Approach for the Identification of Novel Metabolites of Ketamine by Mass Spectrometry. Drug Metab Dispos. 2009;37:1769–1778.

2. Papp M, Gruca P, Lason M, Litwa E, Solecki W, Willner P. AMPA receptors mediate the pro-cognitive effects of electrical and optogenetic stimulation of the medial prefrontal cortex in antidepressant non-responsive Wistar–Kyoto rats. J Psychopharmacol. 2020;34:1418–1430.

3. Kantor S, Lanigan M, Giggins L, Lione L, Magomedova L, de Lannoy I, et al. Ketamine supresses REM sleep and markedly increases EEG gamma oscillations in the Wistar Kyoto rat model of treatment-resistant depression. Behavioural Brain Research. 2023;449:114473.

4. Tallon-Baudry C, Bertrand O, Delpuech C, Pernier J. Oscillatory γ-Band (30 –70 Hz) Activity Induced by a Visual Search Task in Humans. J Neurosci. 1997;17:722–734.
